# Alternative Splicing Events and Splicing Factors Are Prognostic in Adrenocortical Carcinoma

**DOI:** 10.3389/fgene.2020.00918

**Published:** 2020-09-03

**Authors:** Jian Lv, Yuan He, Lili Li, Zhihua Wang

**Affiliations:** ^1^Central Laboratory, Renmin Hospital of Wuhan University, Wuhan, China; ^2^Department of Cardiology, Renmin Hospital of Wuhan University, Wuhan, China

**Keywords:** alternative splicing, Adrenocortical carcinoma, splicing factor, prognosis, hub gene

## Abstract

Alternative splicing is involved in the pathogenesis of human diseases, including cancer. Here, we investigated the potential application of alternative splicing events (ASEs) and splicing factors (SFs) in the prognosis of adrenocortical carcinoma (ACC). Transcriptome data from 79 ACC cases were downloaded from The Cancer Genome Atlas database, and percent spliced-in values of seven splicing types were downloaded from The Cancer Genome Atlas SpliceSeq database. By the univariate Cox regression analysis, 1,839 survival-related ASEs were identified. Prognostic indices based on seven types of survival-related ASEs were calculated by multivariate Cox regression analysis. Survival curves and receiver operating characteristic curves were used to assess the diagnostic value of the prognostic model. Independent prognosis analysis identified several ASEs (e.g., THNSL2| 54469| ME) that could be used as biomarkers to predict the prognosis of patients with ACC accurately. By analyzing the co-expression correlation between SFs and ASEs, 188 highly correlated interactions were established. From the protein interaction network, we finally screened six hub SFs, including YBX1, SART1, PRCC, SNRPG, SNRPE, and SF3B4, whose expression levels were significantly related to the overall survival and prognosis of ACC. Our findings provide a reliable model for predicting the prognosis of ACC patients based on aberrant alternative splicing patterns.

## Introduction

Alternative splicing of pre-messenger RNA (mRNA) produces transcript isoforms for 95% of human genes, increases protein diversity, and provides functional diversity at various regulation level (Pan et al., 2008). There are seven types of splicing patterns, including alternate acceptor site (AA), alternate donor site (AD), alternate promoter (AP), alternate terminator (AT), exon skip (ES), mutually exclusive exons (ME), and retained intron (RI), as listed in The Cancer Genome Atlas (TCGA) SpliceSeq database (Ryan et al., 2016). Splicing factors (SFs) are involved in the removal of introns to create mature mRNAs, a process catalyzed by a large complex termed spliceosome (Yan et al., 2019). Alterations in SF expression lead to missplicing of key cancer-associated genes (Anczukow and Krainer, 2016; Lee and Abdel-Wahab, 2016). Aberrant alternative splicing events (ASEs) have been frequently observed in cancers and is recognized as an important signature for tumorigenesis and related pathologies, such as initiation and development of cancer (Oltean and Bates, 2014; Chen and Weiss, 2015; Lee and Abdel-Wahab, 2016), cancer metabolism (Kozlovski et al., 2017), cancer immunotherapy (Frankiw et al., 2019), cancer drug resistance (Siegfried and Karni, 2018), and so on.

Adrenocortical carcinoma (ACC) is a rare aggressive tumor with poor prognosis and less than 40% survival rate in 5 years (Jouinot and Bertherat, 2018; Crona and Beuschlein, 2019). Recent studies highlighted that specific molecular signatures could predict the survival and prognosis of ACC patients, which came from genomic approaches, including transcriptome, exome or whole-genome sequencing, chromosome alterations, methylome, and miRnome (Barreau et al., 2013; Patel et al., 2013; Assie et al., 2014; Szabo et al., 2014; Jouinot and Bertherat, 2018). However, only limited studies have focused on the potential roles of alternative splicing patterns in the pathogenesis of ACC. The present study aims to investigate the relationship between seven types of ASEs and SFs with the prognosis of ACC. Our findings provide a new path to identify potential targets for the diagnosis and treatment of ACC.

## Results

### Overview of Alternative Splicing Events in Adrenocortical Carcinoma

**FIGURE 1:**
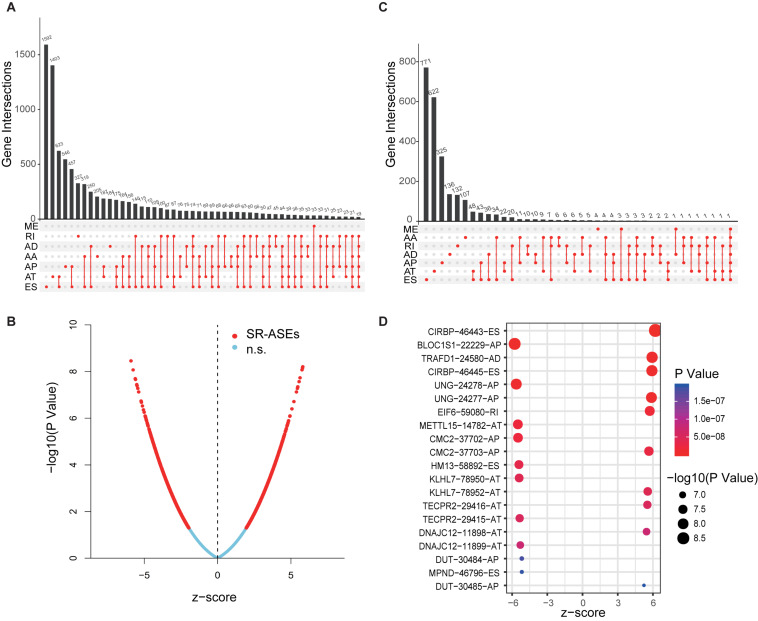
Overview of ASEs and SR-ASEs profiling in ACC. **(A)** UpSet plot of top 50 ASEs with frequency in ACC. **(B)** Volcano plot of 22,521 ASEs. Red points represent the 1,839 SR-ASEs under *P* < 0.01. Transverse axis is the Z score of univariate Cox regression analysis. **(C)** UpSet plot of top 50 SR-ASEs with frequency. **(D)** Bubble plot of the top 20 most significant SR-ASEs. Size of point represents -log10(*P*-value), and the color of points represents *P*-value. Terms of ASEs contain three parts: the gene name, a unique splicing event ID number, and alternative splicing type. AA, alternate acceptor site; AD, alternate donor site; AP, alternate promoter; AT, alternate terminator; ES, exon skip; ME, mutually exclusive exons; RI, retained intron.

Transcriptome data from the TCGA database provide identity information for up to 56,754 transcripts, which represents a key resource for exploring ASEs in tumors concurrently deposited in the database. Individual ASE is assigned a unique annotation in the TCGA SpliceSeq database; for instance, in the term CIRBP| 46443| ES, CIRBP is the official gene symbol, 46443 is the unique ID number of a specific ASE, and ES represents the type of the alternative splicing pattern. Full datasets of splicing events of 79 ACC cases have been deposited in TCGA SpliceSeq, which contains 34,419 ASEs corresponding to 8,994 parent genes. The splicing event data are presented as the percent-spliced-in (PSI), a score to evaluate the level of specific ASEs. The data were firstly filtered by deleting the unreliable ASEs with mean PSI of less than 0.05 and a standard deviation of PSI of less than 0.01. This generated a total of 22,521 ASEs from 8,040 parent genes. As summarized in the UpSet plot (Figure 1A), ES and AT were the top splicing types with high frequency in most genes, whereas ME was the least frequent splicing type.

### Identification of Survival-Related Alternative Splicing Events

**FIGURE 2:**
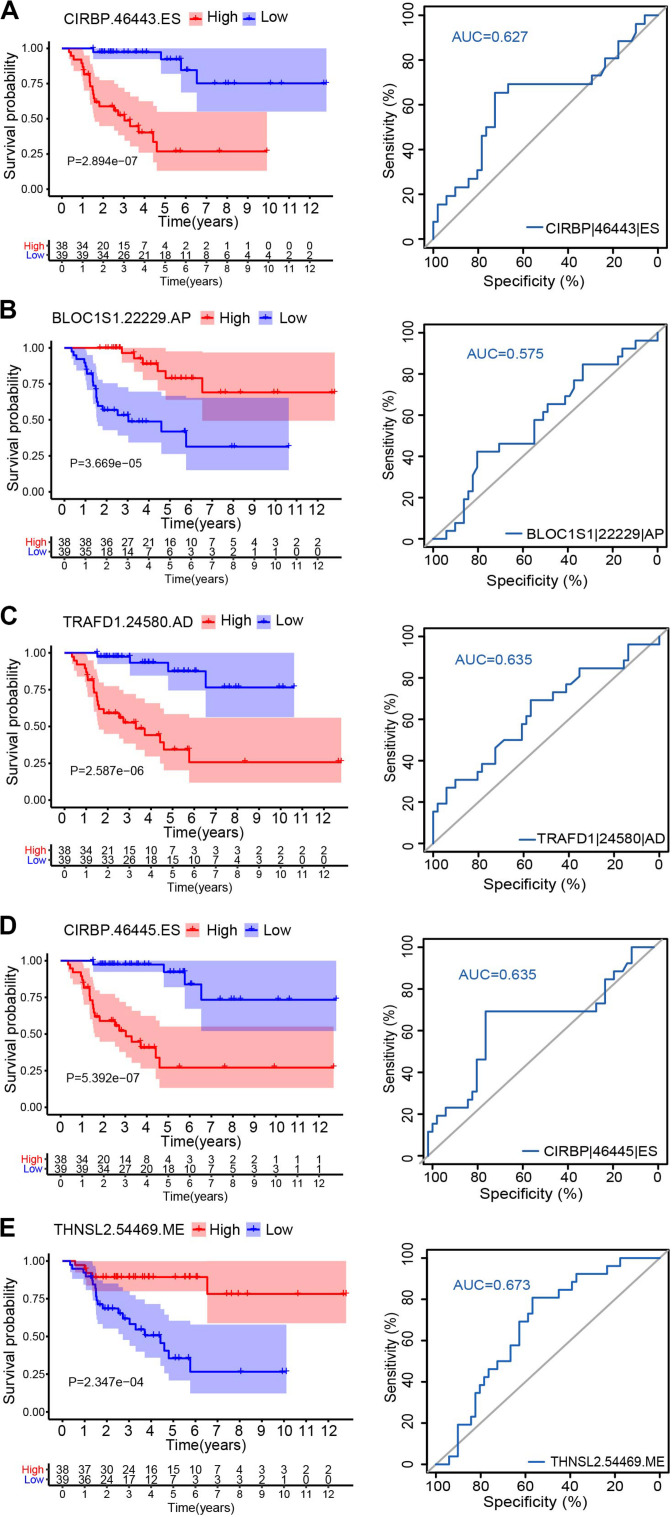
Kaplan–Meier survival curves and ROC curves for five alternative splicing type: CIRBP| 46443| ES **(A)**, BLOC1S1| 22229| AP **(B)**, TRAFD1| 24580| AD **(C)**, CIRBP| 46445| ES **(D)**, and THNSL2| 54469| ME **(E)**. Seventy-nine ACC patients were divided into high- and low-risk groups based on the median of PSI. Red line indicates the high PSI score group, and the blue line indicates the low PSI score group.

To explore the relationship between the alternative splicing pattern and the prognosis of ACC, we performed a univariate Cox regression analysis by comparing ASEs with the overall survival of ACC patients. A total of 1,839 ASEs were significantly associated with overall survival in ACC patients (*P* < 0.01), termed survival-related ASEs (SR-ASEs; Figure 1B). The upSet plot showed that ES, AT, and AP were the most frequent SR-ASEs, whereas only a small number of genes displayed a combination of multiple splicing forms (Figure 1C). Supplementary Figure 1 listed the top 20 most significant SR-ASEs in each splicing pattern according to their Z score and *P-*value. Taking all types together, splicing events within the parent genes CIRBP, BLOC1S1, TRAFD1, UNG, EIF6, METTL15, CMC2, HM13, KLHL7, TECPR2, DNAJC12, DUT, and MPND were highly related to the overall survival of ACC patients (Figure 1D). Survival curves with a cutoff at the median PSI value of the top four ASEs showed a remarkable difference (Figures 2A–D).

### Independent Prognostic Predictors of Survival-Related Alternative Splicing Events in Adrenocortical Carcinoma

**FIGURE 3:**
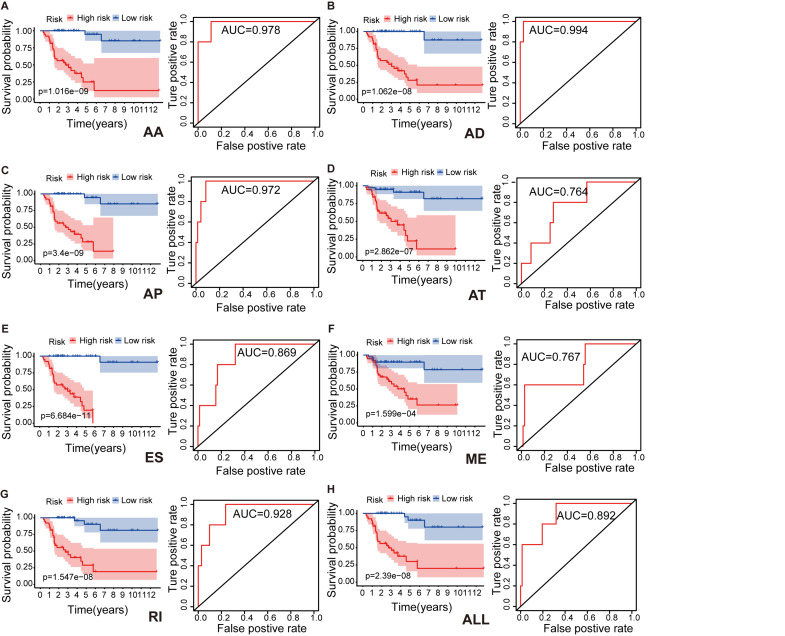
Kaplan–Meier survival curves and ROC curves for each alternative splicing type: AA **(A)**, AD **(B)**, AP **(C)**, AT **(D)**, ES **(E)**, ME **(F)**, RI **(G)**, and ALL **(H)** model. Seventy-nine ACC patients were divided into high- and low-risk groups based on the median of prognostic indices. Red line indicates the high-risk group, and blue line indicates the low-risk group. Prediction time of ROC curve is 1 year. AA, alternate acceptor site; AD, alternate donor site; AP, alternate promoter; AT, alternate terminator; ES, exon skip; ME, mutually exclusive exons; RI, retained intron.

To identify independent prognostic indices and to explore the relationship between aberrant types of ASEs and ACC survival outcomes, we performed the multivariate Cox regression analysis for each splicing type to build a prognostic model. Lasso regression analysis was done to select the most significant SR-ASEs by avoiding overfitting. The median value of risk scores was then used to stratify the 79 ACC patients into low- and high-risk groups. Kaplan–Meier method was used to analyze the efficacy of the prognostic indices to predict the overall survival. The plotted survival curves and receiver operating characteristic (ROC) curves were shown in Figure 3. Significant differences in survival curves were observed in individual splicing type as well as all together (ALL), indicating that each alternative splicing type could be recognized as an independent prognostic indicator (Figure 3). The greatest difference in overall survival curves was observed in ES type (*P* = 6.684e-11), which is the most frequent splicing type among ASEs and SR-ASEs (Figures 1A,C). The area under the curve (AUC) of each ROC curve was more than 0.7, indicating the predictive efficiency of the eight models. We found that the AUC for AA type is 0.978, which is higher than all others (Figure 3), indicating that the prognostic indices based on AA type demonstrated the greatest efficacy in stratifying patients.

We also performed multivariate Cox regression analysis to evaluate the effect of other clinical parameters, including sex, tumor stage, T and N stages in the tumor–node–metastasis (TNM) classification in Table 1. The significant hazard ratios (HRs) for T stage and tumor stage were 3.349 (95% confidence interval [CI]: 2.075–5.403) and 2.886 (CI: 1.819–4.579), respectively (Table 1). HRs of AA, AD, AP, AT, ES, RI, and ALL models are also shown in Table 1. Among the seven types, the HR for ME type was 1.270 (CI: 1.093–1.476), the highest HR value (Table 1).

**TABLE 1:** Univariate Cox regression analysis of AA, AD, AP, AT, ES, ME, RI, and ALL models.

Term	Hazard ratio	HR.95L^#^	HR.95H^#^	*P*-value
Sex	0.963	0.425	2.181	0.928
Tumor stage	2.886	1.819	4.579	6.75E-06
T	3.349	2.075	5.403	7.36E-07
N	2.088	0.781	5.578	0.142
AA	1.003	1.001	1.004	0.001
AD	1.007	1.003	1.011	6.17E-04
AP	1.018	1.010	1.026	3.29E-06
AT	1.095	1.060	1.130	2.29E-08
ES	1.009	1.005	1.012	8.51E-07
ME	1.270	1.093	1.476	0.002
RI	1.009	1.005	1.012	3.18E-06
ALL	1.014	1.007	1.021	4.89E-05

*^#^HR.95L and HR.95H: 95% confidence interval.*

**FIGURE 4:**
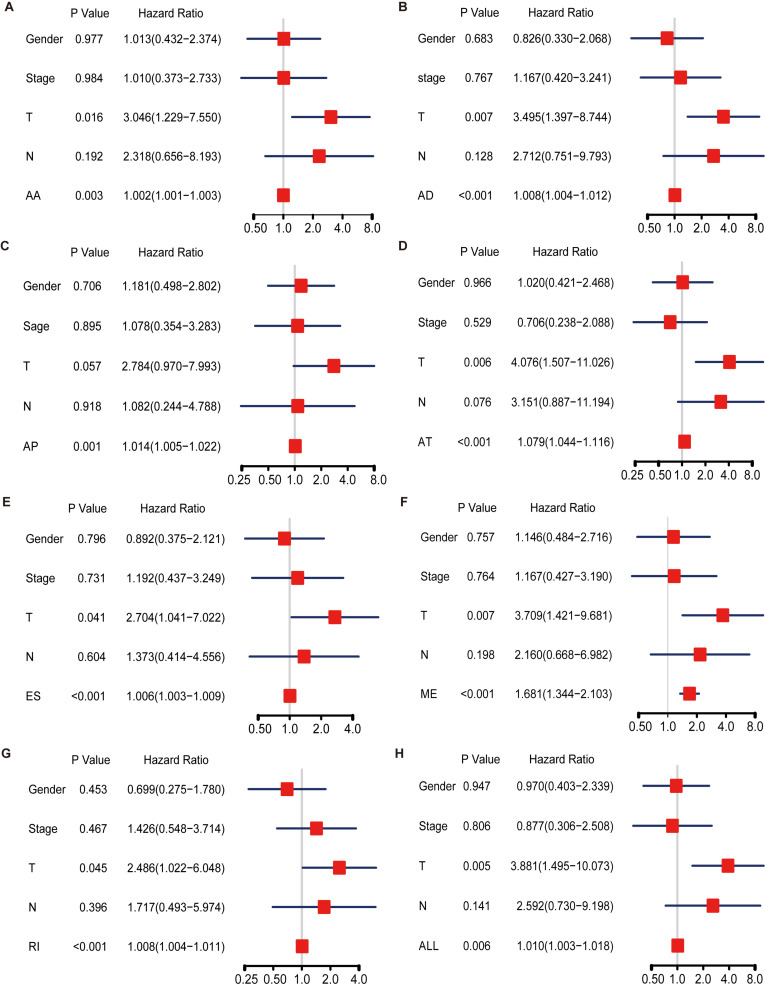
Multivariate Cox regression analysis for AA **(A)**, AD **(B)**, AP **(C)**, AT **(D)**, ES **(E)**, ME **(F)**, RI **(G)**, and ALL **(H)** model. Hazard ratio is shown as hazard ratio (95% confidence interval). AA, alternate acceptor site; AD, alternate donor site; AP, alternate promoter; AT, alternate terminator; ES, exon skip; ME, mutually exclusive exons; RI, retained intron.

Hazard ratios in each of AA, AD, AP, AT, ES, RI, and ALL models with clinical parameters are shown in Figure 4. The HRs for all the AS type were under a significant level (*P-*value < 0.05), and ME type has the highest HR value of 1.681 (CI: 1.344–2.103) (Figure 4F). Moreover, THNSL2| 54469| ME ranks that most significant event in the ME type (Supplementary Figure 1F). THNSL2| 54469| ME was the top significant SR-ASE in ME model to predict the prognostic status of ACC cases. Therefore, the most significant SR-ASEs in ME pattern is THNSL2| 54469| ME (Figure 2E). To test the accuracy of ME model of THNSL2| 54469| ME, the survival state of ACC patients could be significantly classified into high- and low-PSI groups according to the median value of PSI scores of THNSL2| 54469| ME (Figure 2E). These results indicate that THNSL2| 54469| ME could be the best independent prognostic indicator to predict the prognosis of ACC cases.

**FIGURE 5:**
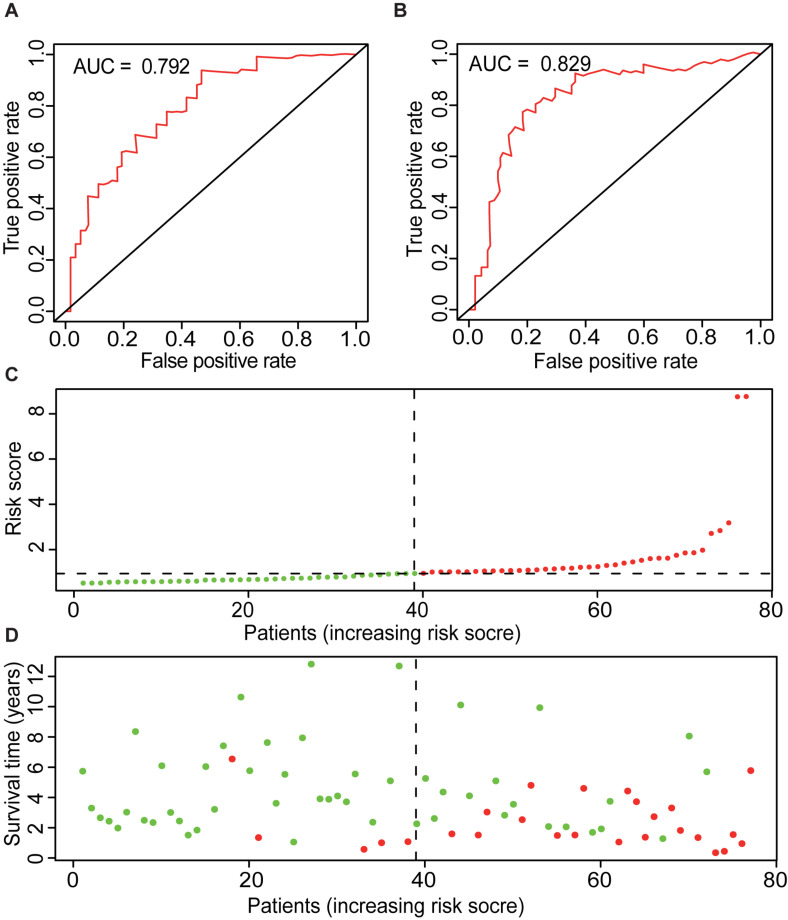
Overview of ME model. ROC curves of ME type with prediction time of 3 years **(A)** and 5 years **(B)**. **(C)** Risk factors of ME model of 79 ACC cases. Patients were divided into low- and high-risk groups based on the median of prognostic indices. Red indicates high-risk group, and green indicates low-risk group. **(D)** Survival time and survival status of ME model of 79 ACC cases. Red indicates the deaths of the cases. Green indicates cases who are alive.

Also, the AUCs for 1, 3, and 5 years of ME model are predicted to be 0.767 (Figure 3F), 0.792 (Figure 5A), and 0.829 (Figure 5B), respectively. The number of deaths increased in the high-risk group, with short survival time (Figures 5C,D). In summary, those results indicated that the constructed ME model had great efficacy in predicting the prognosis of ACC patients.

### Enrichment Analysis of Survival-Related Alternative Splicing Events

**FIGURE 6:**
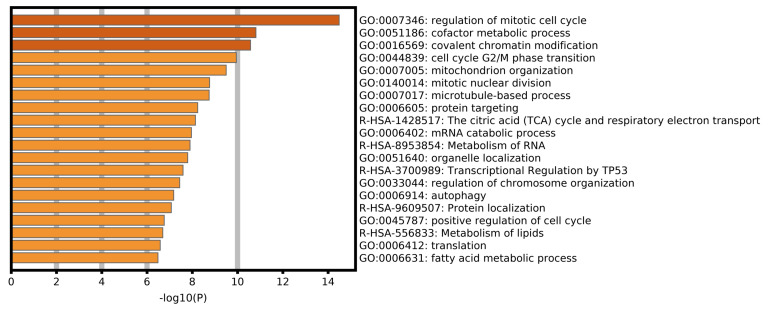
Bar graph of enriched terms across parent genes of SR-ASEs by Metascape, colored by *P*-values.

To explore the biological processes and signal pathways related to alternative splicing in the progression of ACC, enrichment analysis of the SR-ASEs parent genes was performed by gene ontology and pathway analysis in Metascape. The most significantly enriched terms were regulation of mitotic cell cycle, cofactor metabolic process, covalent chromatin modification, cell cycle G2/M phase transition, and mitochondrion organization (Figure 6), pathways that are all involved in tumorigenesis.

### Correlation Network of Survival-Related Alternative Splicing Events and Splicing Factors Expression

Alteration of ASEs is largely attributable to changes in SF expression. We then extracted the expression data of 404 SFs, summarized by a previous study (Seiler et al., 2018), from the transcriptome data of the 79 ACC patients. Principal component analysis (PCA) plots could discriminate the distribution pattern of SF expression levels according to survival state, tumor stage, and TNM classification (Supplementary Figure 2), suggesting an impact of altered SF expression on the ACC outcome.

**FIGURE 7:**
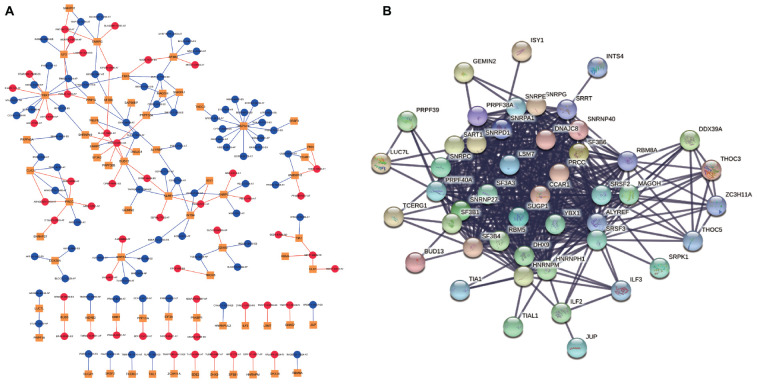
Correlation network of SR-ASEs and SFs. **(A)** Co-expression networks between parent genes of SR-ASEs and SFs. Yellow square indicates SFs, red circle indicates the poor prognostic SR-ASEs, and blue circle indicates the better prognostic SR-ASEs. Red line indicates that SFs were positively correlated with the SR-ASE, and blue line indicates SFs were negatively correlated with the SR-ASE. **(B)** Protein–protein interaction network of SFs.

As our current knowledge is incapable of dissecting the sequence specificity for each SF, we could not establish a direct network for SF-regulated ASEs. Thus, we analyzed the relationship between SFs and SR-ASEs based on their co-expression patterns using the Spearman method, which has been widely used in alternative splicing studies (He et al., 2018; Xiong et al., 2018; Zhang et al., 2020). A total of 188 highly correlated interactions between SFs and SR-ASEs were detected with a correlation coefficient larger than 0.65 (Figure 7A and Table 2). Hub SFs with no less than five SR-ASE connections were HSPA1B, YBX1, SRPK1, SART1, PRCC, ILF2, SNRPG, SNRPE, SF3B4, BUD13, INTS4, and CLK2 (Table 3). Among these SFs, interestingly, SNRPE was exclusively correlated with AT-type ASEs, suggesting a specific role in regulating terminal exon selection (Figure 7A). Moreover, HSPA1B was exclusively correlated with detrimental ASEs showing negative correlations with overall survival, indicating a potential causal role of HSPA1B in the progression of ACC (Figure 7A).

**TABLE 2:** Top 20 significant associations between SR-ASEs and SFs.

Splicing factor	ASEs^#^	Correlation	*P*-value	Regulation
HSPA1B	KCNJ5-19431-AP	−0.84181986	8.92E-22	Negative
HSPA1B	UBE3A-29716-AP	−0.82743598	1.77E-20	Negative
MAGOH	ZNF131-71936-ES	−0.81209442	3.20E-19	Negative
HSPA1B	IKBKB-83691-ES	−0.80904662	5.51E-19	Negative
HSPA1B	CYTH1-43892-ES	−0.80356862	1.43E-18	Negative
HSPA1B	EYS-76614-AT	−0.79837952	3.43E-18	Negative
PRPF38A	ZNF131-71936-ES	−0.78822643	1.77E-17	Negative
SF3B4	ZNF131-71936-ES	−0.788164	1.79E-17	Negative
HSPA1B	GNG7-46614-AP	−0.77278859	1.83E-16	Negative
PRPF38A	ZNF131-71932-ES	−0.77084258	2.42E-16	Negative
BUD13	TNFRSF12A-33348-ES	−0.77031719	2.61E-16	Negative
PRPF38A	F6-23309-ES	−0.75603278	1.88E-15	Negative
PRPF38A	HDDC3-32524-AA	0.751788406	3.30E-15	Positive
SNRPA1	ZNF131-71936-ES	−0.74748153	5.77E-15	Negative
HSPA1B	DAGLB-78732-ES	−0.74496838	7.96E-15	Negative
SRPK1	NDUFA12-23740-ES	−0.74060614	1.38E-14	Negative
SNRPG	KIF20B-12497-AT	−0.73362818	3.23E-14	Negative
SNRPG	KIF20B-12498-AT	0.733628184	3.23E-14	Positive
PRCC	ARHGEF28-72492-AT	−0.73325406	3.38E-14	Negative
PRCC	ARHGEF28-72493-AT	0.733254055	3.38E-14	Positive

*^#^In terms of KCNJ5-19431-AP, KCNJ5 is an official gene symbol, 19431 is the number of alternative splicing events, and AP is the type of AS pattern.*

**TABLE 3:** Two MCODE modules of protein–protein interaction network of parent genes of SR-ASEs and SFs.

Module	Score	Gene symbol
Module 1	27	SART1, SNRPA1, SNRPG, SF3B1, SF3B6, SNRPD1, RBM8A, MAGOH, SNRPE, SRSF3, LSM7, SF3B4, SF3A3, SRSF2, HNRNPM, SNRPC, SNRNP40, HNRNPH1, SRRT, ALYREF, PRPF38A, PRPF40A, DHX9, YBX1, SNRNP27, RBM5, DNAJC8, CCAR1, PRCC, SUGP1
Module 2	8	THOC5, THOC3, DDX39A, ZC3H11A

**FIGURE 8:**
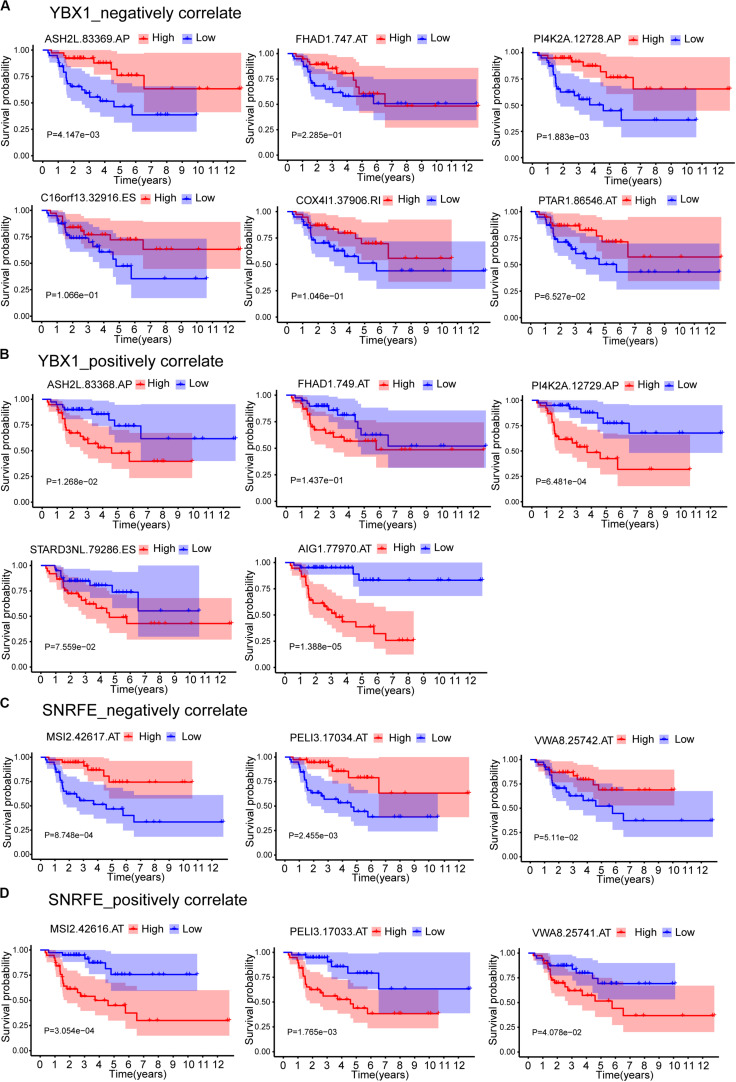
Kaplan–Meier survival curves for ASEs that correlated with splicing factors YBX1 and SNRFE. **(A)** ASEs that negatively correlated with YBX1. **(B)** ASEs that positively correlated with YBX1. **(C)** ASEs that negatively correlated with SNRFE. **(D)** ASEs that positively correlated with SNRFE. Seventy-nine ACC patients were divided into high- and low-risk groups based on the median of PSI. Red line indicates the high PSI score group, and blue line indicates the low PSI score group.

The expression data showed that SF YBX1 was positively correlated with the PSI values of C16orf13| 32916| ES, COX4I1| 37906| RI, FHAD1| 747| AT, MYL6| 22381| AA, PI4K2A| 12728| AP, ASH2L| 83369| AP, PTAR1| 86546| AT, and IRF3| 51012| ES and was negatively correlated with the PSI values of FHAD1| 749| AT, AIG1| 77970| AT, PI4K2A| 12729| AP, STARD3NL| 79286| ES, and ASH2L| 83368| AP; SF SNRFE was positively correlated with the PSI values of VWA8| 25742| AT, MSI2| 42617| AT, and PELI3| 17034| AT and was negatively correlated with the PSI values of VWA8| 25741| AT, MSI2| 42616| AT, and PELI3| 17033| AT (Figure 7A). We then constructed the Kaplan–Meier survival curves for each ASEs related with YBX1 and SNRFE and found that high levels of PSI values for all the negatively correlated ASEs have a better prognostic except FHAD1| 747| AT, whereas the low levels of PSI values for all the positively correlated ASEs have a better prognostic (Figure 8). Interestingly, six pairs of ASEs from three parent genes were observed for these two SFs in the network. For example, ASH2L| 83368| AP and ASH2L| 83369| AP are two alternative splicing sites for ASH2L exon 1 selection. PI4K2A| 12728| AP and PI4K2A| 12729| AP are two alternative splicing sites for ASH2L exon 1 selection (Figures 8A,B). Our results indicate that promoter selections of ASH2L and PI4K2A are important for tumorigenesis of ACC. Similar results were also observed for the SF SNRFE (Figures 8C,D). These results suggest that terminator selections of MSI2 and PELI3 and VWA8 are important for the progression of ACC development (Figures 8C,D).

Considering that SFs would influence each other when regulating the work mode of the spliceosome, we constructed the protein–protein internecion network to illustrate the interactions among SFs using the Search Tool for the Retrieval of Interacting Genes database (Figure 7B). A total of 66 nodes and 485 edges were revealed, with the interaction score of 0.900 (Figure 7B). Module analysis was done to identify hub genes. Two modules were further identified by the app MCODE in Cytoscape. Module 1 has 30 genes with a score of 27, and module 2 has four genes with a score of 8 (Table 3). By combining the ASE correlation results and protein interaction networks, we finally identified six hub SFs, including YBX1, SART1, PRCC, SNRPG, SNRPE, and SF3B4 (Table 4). Overall, these results indicate that these six hub genes may play an important role in the progression of ACC by regulating the pattern of SR-ASEs.

**TABLE 4:** Counts of SFs correlated with SR-ASEs in correlation network.

Splicing factor	Count
HSPA1B	14
YBX1	13
SRPK1	7
SART1	7
PRCC	7
ILF2	7
SNRPG	6
SNRPE	6
SF3B4	6
BUD13	6
INTS4	5
CLK2	5

### Analysis of Hub Splicing Factors

**FIGURE 9:**
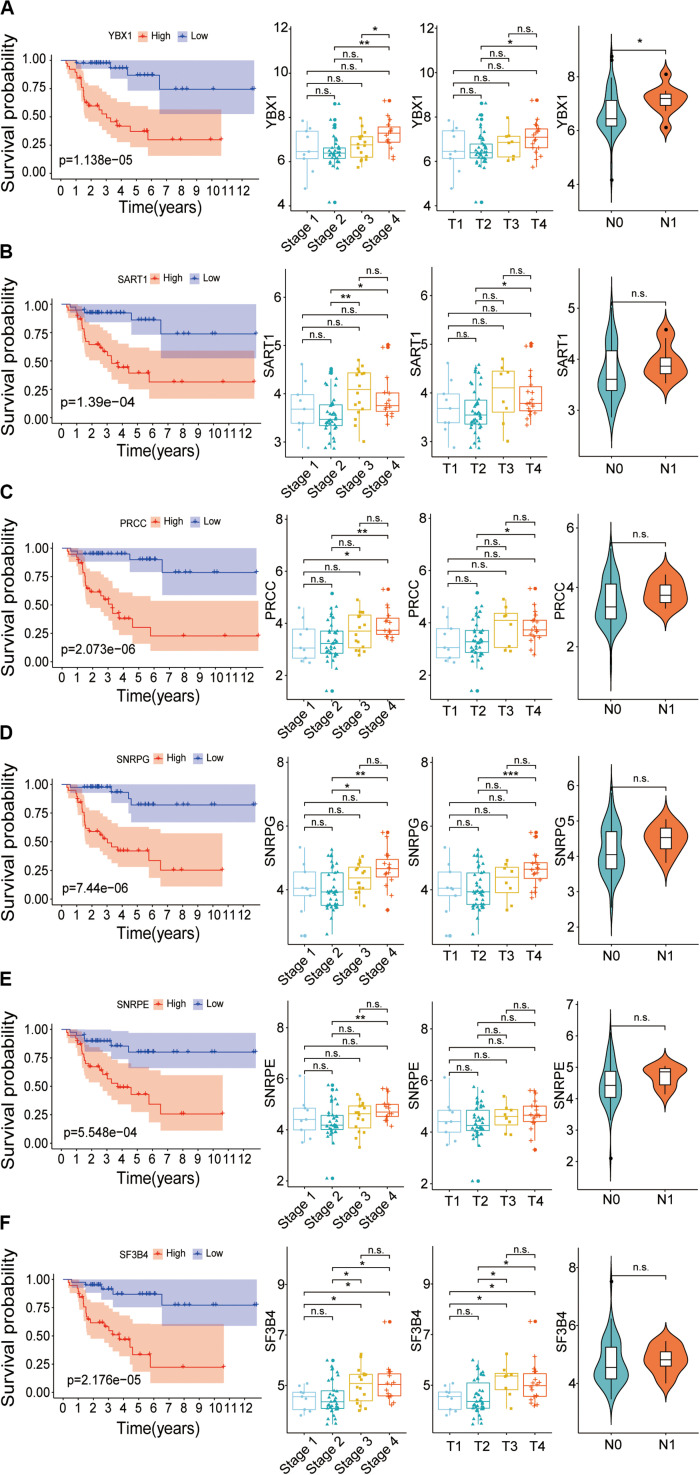
Relationship between hub SFs and survival curve, tumor stage, T stage and lymph node state. **(A)** YBX1. **(B)** SART1. **(C)** PRCC. **(D)** SNRPG. **(E)** SNRPE. **(F)** SF3B4. *P < 0.05; **P < 0.01; ***P < 0.001 and n.s. indicates no significance.

To confirm whether these six genes, YBX1, SART1, PRCC, SNRPG, SNRPE, and SF3B4, are high-risk factors, the overall survival and expression level of these genes were further investigated. The results showed that the survival rate of patients with high expression of hub SFs was significantly lower than that in the low expression group (*P* < 0.001) (Figure 9), and the results were consistent with those from the Gene Expression Profiling Interactive Analysis (GEPIA) database (Supplementary Figure 3).

The correlation between the expression levels of six SFs and clinical information, including clinical stages, tumor stage, and lymph nodes stage, was further analyzed. We found that the expression levels of SFs increased along with the clinical stages. The expression level of six hub SFs in tumor patients has an increasing trend with the tumor progress stage. Among six SFs, the level of SF3B4 in ACC cases with the tumor stage III and the level of PRCC and SF3B4 in ACC cases with stage IV were statistically different from those in stage I (Figure 9), which is consistent with the results from the GEPIA database (Supplementary Figure 3). The levels of the SFs in ACC cases with lymph node stage also increased, and the expression of YBX1 was statistically changed. The results indicate that the expression levels of these SFs are closely related to the survival time and prognosis of patients with ACC (Figure 9).

**FIGURE 10:**
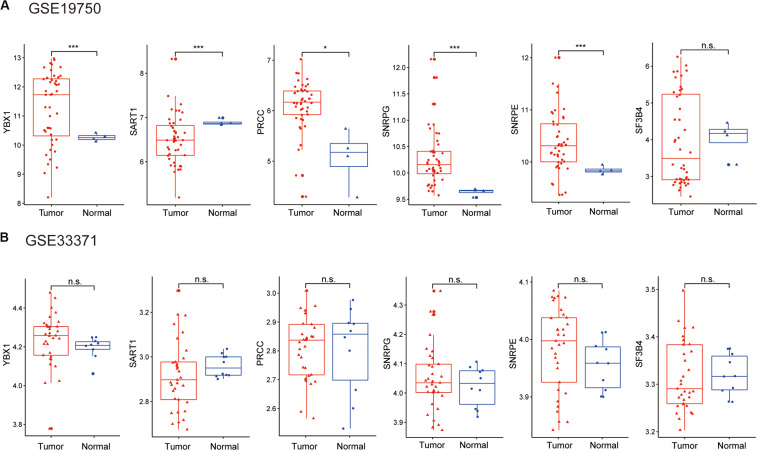
Expression level of hub SFs of ACC or normal samples in the microarray: **(A)** GSE19750 and **(B)** GSE33371. *P < 0.05; ***P < 0.001 and n.s. indicates no significance.

Considering the lack of normal control tissue for ACC in the TCGA database, two microarray datasets from Gene Expression Omnibus (GEO) database were used to compare the expression of SFs between tumor tissues and normal controls (Figure 10). The mRNA levels of YBX1 and SNRPE were increased in tumor samples in the two microarrays, although the expression levels only showed statistically significant differences in GSE19750 (Figure 10). Combining the mRNA level of YBX1 and SNRPE in different tissues and the effects of YBX1 and SNRPE on the survival curve of ACC cases (Figure 7), we could conclude that YBX1 and SNRPE could exert positive regulation in the progression of ACC.

We next performed a Cox regression analysis to evaluate the prognostic value of these six hub SFs and other clinical parameters. Results showed that the HRs of these six genes ranged from 1.003 to 1.156; although the HR is lower than the T and N stages, it statistically significant for all the six SFs (Supplementary Figure 4). The AUC of each ROC curve is higher than 70% (Supplementary Figure 4), indicating that all of these six SFs could be used to classify the stages of ACC.

## Discussion

Adrenocortical carcinoma is a rare malignancy tumor with a poor prognosis. Currently, surgery is the only available curative treatment option (Crona and Beuschlein, 2019). Recent studies (Barreau et al., 2013; Patel et al., 2013; Assie et al., 2014; Szabo et al., 2014; Jouinot and Bertherat, 2018) highlighted that genomic approaches derived from TCGA database and GEO datasets could provide specific molecular signatures for the diagnosis and prognosis of ACC. At present, few studies focused on the different isoforms of alternative splicing in ACC (Bie et al., 2019). The present study is to explore the aberrant of ASEs and hub SFs to develop novel diagnostic and prognostic markers for ACC.

Seven types of ASEs were investigated in this study. ES type was the top splicing type with high-frequency ASEs and SR-ASEs (Figures 1A,C), indicating the ES is the dominant alternative splicing type in ACC.

In this study, we identified 1,839 SR-ASEs by univariate Cox regression analysis. Interestingly, different ASEs in the same gene could exert opposite functions in the overall survival of ACC, indicating that the parent genes of these ASEs may play an important role in ACC development (Figures 1D, 8). Because ME type has the highest HR value (Figure 4F) THNSL2| 54469| ME ranks the most significant event in the ME type (Supplementary Figure 1F). Therefore, THNSL2| 54469| ME could be used as an independent prognostic indicator to predict the prognosis of ACC cases (Figure 2E). THNSL2 encodes a threonine synthase-like protein and has multiple transcript variants. The function of THNSL2 and the alternative splicing of THNSL2| 54469| ME could be further investigated. Enrichment analysis revealed several important pathways, including regulation of the mitotic cell cycle and cell cycle G2/M phase transition, which could impact the occurrence and development of ACC (Figure 6).

Genes involved in the same biological process or signaling pathway are usually co-regulated in the disease context. Co-expression network analysis has been widely used to dissect the functional gene panels in large datasets, including alternative splicing studies (He et al., 2018; Xiong et al., 2018; Zhang et al., 2020). One hundred eighty-eight highly correlated interactions between SFs and SR-ASEs were identified (Figure 7). SR-ASEs that were positively correlated with SFs have a poor prognostic value, whereas SR-ASEs that were positively correlated with SFs have a poor prognostic value (Figures 7, 8). It provided a new insight for the molecular mechanism of alternative splicing in ACC.

The number of pairs of ASEs from one parent gene was observed for SFs in the network (Figure 7), and six pairs of ASEs related with YBX1 and SNRFE were illustrated in detail (Figure 8). We observed that the pairs of ASEs conferred the opposite function for the ACC progress shown in the overall survival curve (Figure 8), indicating that specific exons were important, such as the alternative promoters’ selection of ASH2L, and alternative terminator selection of MSI2. Moreover, among these six pairs of ASEs, only alternative promoters of ASH2L have been reported in embryonal carcinoma (Alagaratnam et al., 2013).

A previous study on ASEs in endometrial cancer also identified YBX1 as the hub SF. One pair of ASEs that correlated with YBX1 was identified in that study: DNAH9-AT-39292 and DNAH9-AT-39293 (Wang et al., 2019). We could conclude that, firstly, it seems YBX1 more specifically regulates the first exon, as the splicing type is AT type in both studies; secondly, YBX1, as the hub SF, might regulate specific genes in different cancer types.

Six hub SFs, including YBX1, SART1, PRCC, SNRPG, SNRPE, and SF3B4, were identified in this study. We found that the expression level of hub SFs was negatively correlated with the survival time and survival state of ACC patients (Figure 9). There was still some evidence that the expression level of hub SFs in tumor tissues was higher than that in normal tissues (Figure 10). The altered expression level of hub SFs has been reported in multiple types of cancer, such as YBX1 (Rossner et al., 2016; Chen et al., 2019), SART1 (Ishida et al., 2000; Sasatomi et al., 2000; Yutani et al., 2001), SNRPE (Tamura et al., 2007; Jia et al., 2011), and SF3B4 (Liu et al., 2018). Other studies have shown that SNRPE (Quidville et al., 2013) and SF3B4 (Shen and Nam, 2018) could develop a new therapeutic agent in cancer. Further study found that the inactivation of SF3B4 inhibited liver tumorigenesis *in vitro* and *in vivo* (Shen et al., 2018). In terms of molecular mechanism, SF3B4 triggers SF3b complex to splice tumor suppressor KLF4 transcript to non-functional skipped exon transcripts, downregulates the transcriptional activity of p27Kip1, and upregulates the transcriptional activation of Slug genes (Shen et al., 2018). However, the functions of hub SFs in ACC development need to be further studied.

Our present study performed a bioinformatic analysis of SR-ASEs and hub SFs and provided insight into the function of aberrant ASEs in ACC development and progression. SR-ASEs and hub SFs identified in this study could be potential targets for the diagnosis of ACC patients.

## Materials and Methods

### Data Collection

The transcriptome data of 79 ACC cases and the corresponding clinical information, including survival time, survival status, sex, tumor stage, T stage, and lymph node metastasis, were downloaded from TCGA^1^. Seven types of ASEs of 79 ACC cases were downloaded from the TCGA SpliceSeq database^2^ (Ryan et al., 2016). ASEs were quantified using the PSI, rating from 0 to 1. Splicing event data of 79 ACC cases included 34,419 ASEs, corresponding to 8,994 genes. To better track the AS events, the name of the ASEs contains three parts: the official gene symbol, a unique splicing event ID number, and splicing type. The number of ASEs for different types of ASEs are shown in the UpSet plot (Figure 1A).

### Screening of Survival-Related Alternative Splicing Events

The missing value of PSI in seven AS event types was supplemented by impute.knn function using nearest neighbor averaging method with *k* = 10, rowmax = 0.5, colmax = 0.8, and other default setting in R. Then, the PSI data of 79 ACC cases were filtered by deleting the ASEs with mean PSI of less than 0.05 and standard deviation of PSI of less than 0.01. The filtered PSI data, including 22,521 ASEs from 8,040 genes in 79 ACC cases, were used for visualization and subsequent analysis. The survival time and survival status of 79 ACC cases were integrated with the filtered PSI data. Then, SR-ASEs were selected using univariate Cox regression analysis with a threshold set to a *P*-value < 0.01 and were visualized by volcano plot and bubble plot.

### Construction of Prognosis Model of Survival-Related Alternative Splicing Events

Multivariate Cox regression analysis was performed to calculate the prognostic indices of each for each type of splicing pattern. To prevent over-fitting of the model, seven different types of SR-ASEs and ALL SR-ASEs were analyzed by lasso regression analysis, making the Lambda values at smaller level. The SR-ASEs with high correlation have been removed to guarantee the accuracy of the model. Risk factors were calculated using the following formula βSR-ASE1 × PSISR-ASE1 + βSR-ASE2 × PSISR-ASE2 + …… + βSR-ASEn × PSISR-ASEn, where β corresponded to the regression coefficient. The samples were stratified to high- and low-risk groups based on the median of risk scores. The survival curve and ROC curve of the 79 ACC cases were used to evaluate the accuracy of the model. Univariate and multivariate Cox regression analyses were used to determine whether the prognostic indices could be used as an independent prognostic factor for predicting the prognosis of ACC cases.

### Enrichment Analysis of Survival-Related Alternative Splicing Events

To illustrate the biological functions and pathway associated with SR-ASEs of ACC cases, gene enrichment analysis for parent genes of SR-ASEs was performed in the online database Metascape^3^ (Zhou et al., 2019) with the default setting. Metascape is an online analytical tool for pooled gene annotation, enrichment analysis, and protein interaction analysis.

### Construction of Splicing Correlation Network

Four hundred four SFs summarized from the previous study were used in this study for SF analysis (Seiler et al., 2018). They collected and prioritized the final list of 404 SF genes by compiling and filtering spliceosome and splicing related genes from three sources, which were all experimentally validated in the previous study (Barbosa-Morais et al., 2006; Hegele et al., 2012; Cvitkovic and Jurica, 2013). Source 1 (Hegele et al., 2012) was from a comprehensive yeast two-hybrid study using spliceosome components as bait. Source 2 (Barbosa-Morais et al., 2006) included 254 SFs and splicing related proteins. Source 3 was from SpliceosomeDB (Cvitkovic and Jurica, 2013). Of 404 SFs, 390 were extracted from the transcriptome data of 79 ACC cases. Co-expression network analysis calculated by spearman method was used in the construction of alternative splicing regulation networks to screen the important SFs. Cytoscape visualized a highly correlated interaction network with the threshold of correlation coefficient of 0.65.

Also, protein–protein interaction network of SFs was established to find the important SFs in the progression of ACC, using the online database Search Tool for the Retrieval of Interacting Genes^4^ (version 11.0). Then, the hub SFs were selected by the MCODE app of Cytoscape. Six prognostic-related hub SFs for patients with ACC were identified by combining the co-expression networks analysis and protein–protein internecion networks.

### Analysis of Hub Splicing Factors

Correlations of the mRNA expression level of hub SFs with clinical data (survival curve, tumor stage, T stage, and lymph node state) of 79 ACC cases were analyzed. The online database GEPIA (Tang et al., 2017)^5^, which contains the TCGA database with the GTEx database, was further used to evaluate the SFs on clinical features. Also, three gene expression microarray data were downloaded from GEO^6^ for illustrating the expression levels of hub SFs between normal tissue and tumor tissues. GSE19750 (Demeure et al., 2013) includes 44 ACC samples and 4 control samples. GSE33371 (Heaton et al., 2012) includes 33 ACC samples and 10 control samples.

## Data Availability Statement

The transcriptome data of 79 ACC cases and the corresponding clinical information can be downloaded from The Cancer Genome Atlas (TCGA) (https://tcga-data.nci.nih.gov/tcga/). Seven types of alternative splicing events (ASEs) of 79 ACC cases downloaded from TCGA SpliceSeq database (https://bioinformatics.mdanderson.org/TCGASpliceSeq/). Gene expression microarray data GSE19750 and GSE33371 were downloaded from Gene Expression Omnibus (GEO, http://www.ncbi.nlm.nih.gov/geo).

## Author Contributions

ZW and LL conceived and designed the project. JL and YH downloaded and analyzed the data. JL and LL drafted the manuscript. All authors contributed to text revision and discussion.

## Conflict of Interest

The authors declare that the research was conducted in the absence of any commercial or financial relationships that could be construed as a potential conflict of interest.

## Footnotes

1 https://tcga-data.nci.nih.gov/tcga/

2 https://bioinformatics.mdanderson.org/TCGASpliceSeq/

3 http://metascape.org

4 http://string-db.org

5 http://gepia.cancer-pku.cn/index.html

6 http://www.ncbi.nlm.nih.gov/geo
