# Supplementary figures and images for: Alternative Splicing Events and Splicing Factors Are Prognostic in Adrenocortical Carcinoma
Source: Front Genet. 2020 Sep 3;11:918. doi: 10.3389/fgene.2020.00918 (PMC7494975; doi:10.3389/fgene.2020.00918)

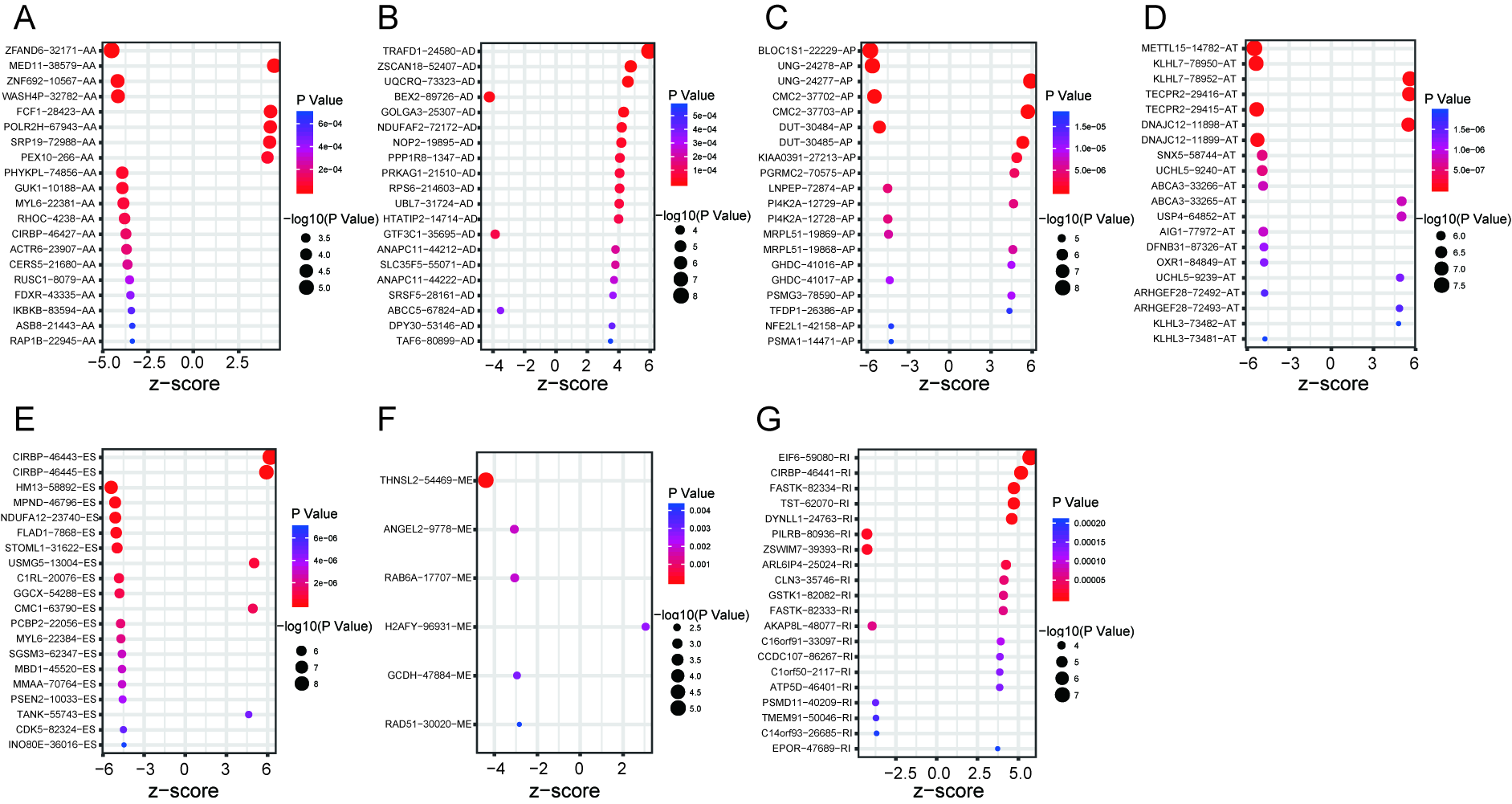

Supplement: Supplementary file 1 [file Image_1.TIF]

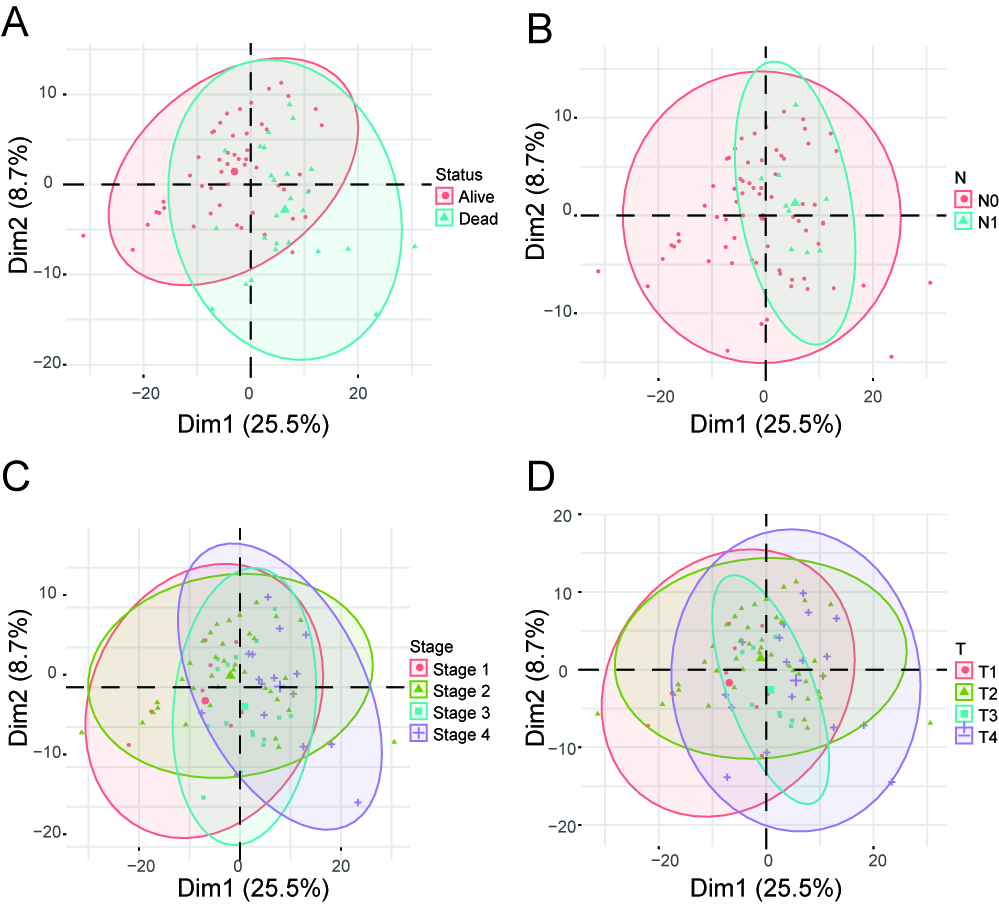

Supplement: Supplementary file 2 [file Image_2.TIF]

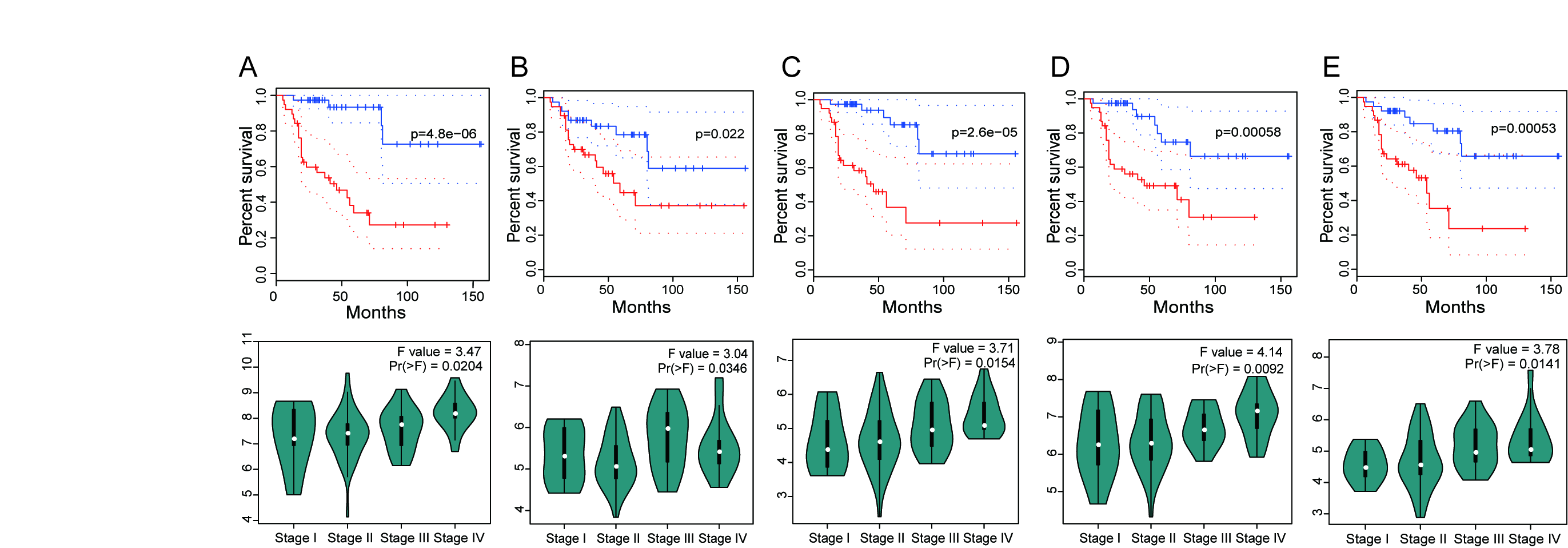

Supplement: Supplementary file 3 [file Image_3.TIF]

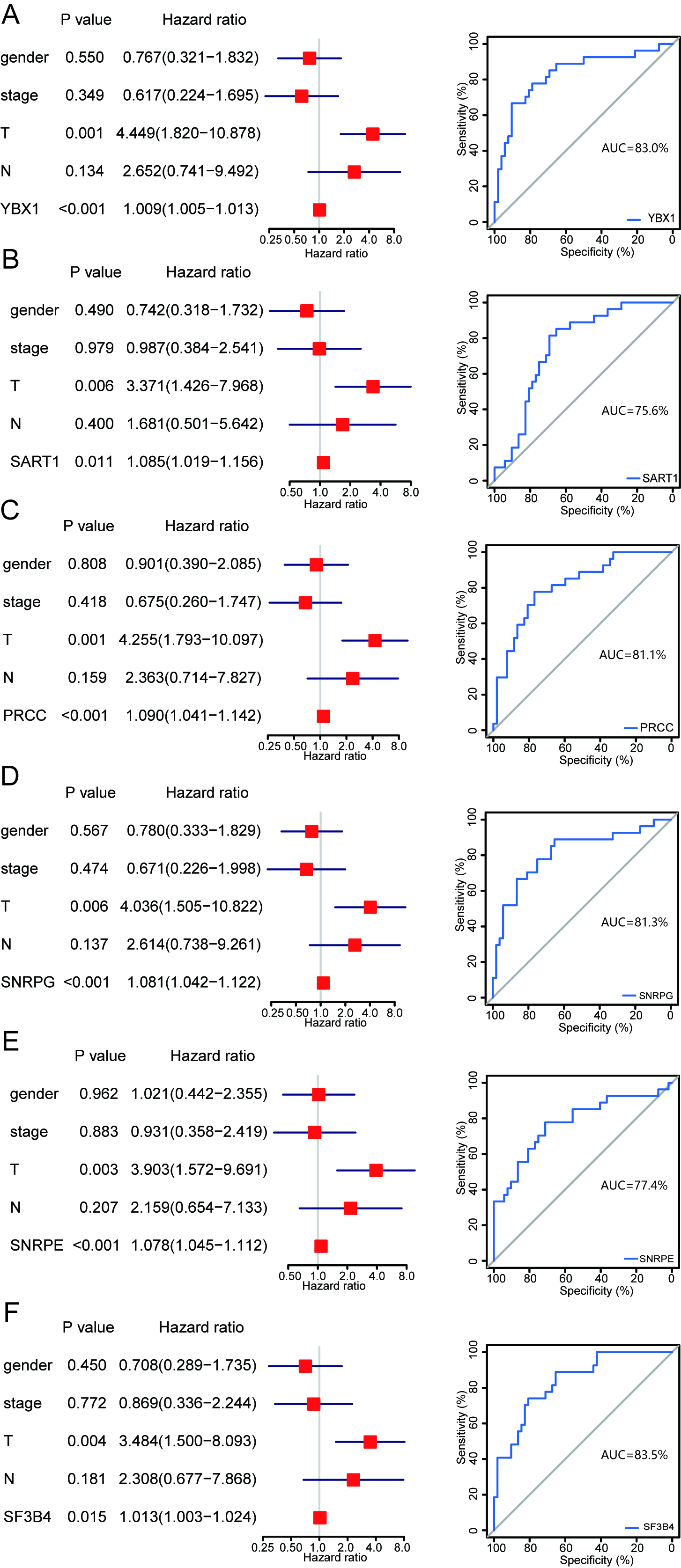

Supplement: Supplementary file 4 [file Image_4.TIF]
