# Supplementary material for: Alternative Splicing Events and Splicing Factors Are Prognostic in Adrenocortical Carcinoma
Source: Front Genet. 2020 Sep 3;11:918. doi: 10.3389/fgene.2020.00918 (PMC7494975; doi:10.3389/fgene.2020.00918)
Supplement: Supplementary file 5 [file Table_1.DOCX]

Supplementary Material

# Supplementary Figures

**Supplementary Figure 1.** Bubble plot of the 20 most significant SR-ASEs in seven AS types. (A) AA, (B) AD, (C) AP, (D) AT, (E) ES, (F) ME, (G) RI.

**Supplementary Figure 2.** Principal component analysis (PCA) plots showing distribution pattern of SFs expression levels according to survival state (A), tumor stage (B) and TNM classification (C and D).

**Supplementary Figure 3.** Relationship between hub SFs and survival curve and tumor stage analyzed in GEPIA database. (A) YBX1. (B) SART1. (C) PRCC. (D) SNRPG. (E) SF3B4.

**Supplementary Figure 4.** Multivariate Cox regression analysis results for 6 hub splicing factors. (A) YBX1. (B) SART1. (C) PRCC. (D) SNRPG. (E) SNRPE. (F) SF3B4. Hazard ratio is shown as hazard ratio (95% confidence interval).
